# Novel Therapies for Relapsed and Refractory Neuroblastoma

**DOI:** 10.3390/children5110148

**Published:** 2018-10-31

**Authors:** Peter E. Zage

**Affiliations:** 1 Department of Pediatrics, Division of Hematology-Oncology, University of California San Diego, La Jolla, CA 92093, USA; pzage@ucsd.edu; Tel.: +1-858-534-6494; 2 Peckham Center for Cancer and Blood Disorders, Rady Children’s Hospital, San Diego, CA 92123, USA

**Keywords:** neuroblastoma, relapsed neuroblastoma, recurrent neuroblastoma, refractory neuroblastoma, personalized treatment, ALK, RET, MIBG, retinoic acid

## Abstract

While recent increases in our understanding of the biology of neuroblastoma have allowed for more precise risk stratification and improved outcomes for many patients, children with high-risk neuroblastoma continue to suffer from frequent disease relapse, and despite recent advances in our understanding of neuroblastoma pathogenesis, the outcomes for children with relapsed neuroblastoma remain poor. These children with relapsed neuroblastoma, therefore, continue to need novel treatment strategies based on a better understanding of neuroblastoma biology to improve outcomes. The discovery of new tumor targets and the development of novel antibody- and cell-mediated immunotherapy agents have led to a large number of clinical trials for children with relapsed neuroblastoma, and additional clinical trials using molecular and genetic tumor profiling to target tumor-specific aberrations are ongoing. Combinations of these new therapeutic modalities with current treatment regimens will likely be needed to improve the outcomes of children with relapsed and refractory neuroblastoma.

## 1. Introduction

With new treatment strategies, including immunotherapy and therapies directed against tumor-specific molecular aberrations, the outcomes of children with high-risk neuroblastoma have significantly improved over the past two decades. Despite these recent successes, however, many of these children continue to suffer from refractory or relapsed disease, and to date, there are no established curative treatment options for many of these patients. Relapsed neuroblastoma has been considered invariably fatal in the past [1], with a median overall survival time for patients with refractory neuroblastoma of only 27.9 months and with an overall survival of only 11.0 months for patients with relapsed disease [2]. However, the reported 5-year overall survival rate for children after the initial relapse of neuroblastoma is 20% [3,4]. These outcomes were dependent on both the time of relapse and the initial patient tumor stage [3,4,5], suggesting that a subset of patients with recurrent neuroblastoma can be cured with appropriate treatment and also emphasizing the need for additional treatment options for these patients.

Recent data presented by Modak and colleagues and Manole and colleagues indicate that patients with isolated local recurrence can be treated successfully with surgical tumor resection and/or radiation therapy at sites of active disease [6,7]. These authors further recommend using chemotherapy regimens with demonstrated activity in children with recurrent neuroblastoma, rather than experimental therapy for localized relapses. However, children with neuroblastoma who suffer disease relapse often develop metastatic tumors resistant to standard therapies, and the underlying molecular profiles and signaling pathway activity in these relapsed tumors are likely significantly different from those in untreated tumors due to novel molecular and genetic aberrations induced or selected by prior therapy [8,9]. The goals of treatment in these patients historically have not been curative, but rather to prolong survival and minimize the toxicities of additional therapy. However, recent data have suggested that more aggressive treatment of these patients to achieve a complete or nearly complete response, followed by additional consolidation or maintenance therapy, can lead to durable disease remission and even cure [10], suggesting that the goals of treatment for many children with relapsed and refractory neuroblastoma may need to be modified.

## 2. Systemic Chemotherapy and Radiotherapy

### 2.1. Systemic Chemotherapy

Initial treatment regimens for patients with relapsed or refractory neuroblastoma typically include chemotherapy combinations distinct from those previously used. Topotecan and irinotecan have both demonstrated activity as single agents in preclinical and clinical studies of neuroblastoma via inhibition of the topoisomerase I enzyme [11,12,13,14,15,16] and are both commonly used for the treatment of children with relapsed neuroblastoma. An early phase II study using a combination of topotecan and cyclophosphamide in children with relapsed neuroblastoma demonstrated objective responses in 6 of 13 patients [17]. A follow-up study demonstrated that children with recurrent neuroblastoma who were treated with the combination of cyclophosphamide and topotecan had increased response rates compared to those who received topotecan as a single agent, although no difference in overall survival rates was observed in treated patients [18,19]. Using a combination of higher doses of cyclophosphamide and topotecan with vincristine, children with primary refractory neuroblastoma had an overall response rate of 19%, while the response rate was 52% for those with their initial neuroblastoma relapse [20]. A phase II study of topotecan combined with doxorubicin and vincristine (TVD) demonstrated a 64% overall response rate in 25 patients with relapsed neuroblastoma, with 4 patients having complete responses to the TVD combination [21]. The TVD regimen was subsequently incorporated into the SIOPEN HR-NBL-1 treatment protocol for high-risk neuroblastoma as salvage therapy for refractory patients with insufficient responses to induction therapy. Following two courses of TVD, 4 of 63 patients (6.4%) enrolled on this protocol had an overall complete response (CR), while 28 of 63 (44.4%) had a partial response (PR). Of note, 23 patients achieved sufficient response to be eligible to receive myeloablative therapy with stem cell rescue [22]. However, the long-term benefits of TVD in these patients will likely need to be established in further clinical trials.

Combinations of irinotecan with the alkylating agent temozolomide have also demonstrated efficacy in children with relapsed neuroblastoma. A single-institution study reported 2 complete responses to the combination of irinotecan and temozolomide among 19 patients with refractory disease, with 7 patients with mixed responses and 10 with stable disease (SD) [23]. Children with relapsed neuroblastoma treated on a separate multi-institutional study using lower doses of irinotecan and temozolomide had an overall response rate of 15%, while an additional 53% had SD [24]. Subsequent clinical trials have attempted to improve on these results with the addition of bevacizumab [25] and temsirolimus [26] to the irinotecan/temozolomide combination. Although these combinations were well tolerated by patients, the additional agents unfortunately did not significantly improve patient outcomes. A subsequent phase I study utilized irinotecan alone combined with the proteasome inhibitor bortezomib, and 2 of 17 evaluable children with refractory or relapsed neuroblastoma demonstrated responses (including one CR), and another 4 children had prolonged stable disease [27], suggesting that this combination might prove more effective in children with relapsed neuroblastoma and should be tested in further studies.

Combinations of ifosfamide, carboplatin, and etoposide (ICE) have also been routinely employed for children with relapsed neuroblastoma. In a single institution study, ICE was well tolerated and led to disease regression in 14 of 17 patients (82%) who were treated after their first neuroblastoma relapse. Disease regression was also seen in 13 of 26 patients (50%) with refractory neuroblastoma and in 12 of 34 patients (35%) who developed progressive disease during frontline treatment [28]. In a separate study, 37% of patients with relapsed or refractory neuroblastoma responded to the ICE regimen, while an additional 17% had SD [29]. Additionally, 15 of 16 children with high-risk neuroblastoma receiving ICE for upfront treatment had either partial or complete responses [30], further demonstrating the efficacy of ifosfamide-based chemotherapy regimens for both frontline treatment of high-risk neuroblastoma and for initial treatment of patients with relapsed neuroblastoma.

Prior studies have also suggested that some patients with relapsed or refractory neuroblastoma might benefit from more aggressive treatment consisting of myeloablative chemotherapy followed by autologous stem cell rescue (ASCR). One recent study demonstrated that children with relapsed neuroblastoma who received myeloablative therapy followed by ASCR had improved survival rates compared to those who received chemotherapy alone (43.5% vs. 9.6%), with 7 of 23 patients achieving either CR or PR after ASCR. However, 2 of the 23 patients experienced transplant-related mortality, suggesting that further studies to identify which patients would receive the most benefit from this approach are needed [31]. Additional studies have suggested that haploidentical stem cell transplantation may also be beneficial for children with relapsed and refractory neuroblastoma, with 5-year event-free survival (EFS) and overall survival (OS) rates of 19% and 23%, respectively, and no treatment-related mortality [32]. However, patients with residual disease who underwent these transplants had significantly worse outcomes than those in complete remission, and a large number of patients experienced acute or chronic graft-versus-host disease (GVHD), suggesting that further study is required.

### 2.2. Radiotherapy

Additional forms of therapy for children with relapsed and refractory neuroblastoma have directly targeted markers expressed on the surfaces of neuroblastoma tumor cells. The vast majority of neuroblastoma tumors demonstrate tumor cell surface expression of the norepinephrine transporter (NET), suggesting that the use of benzylguanidine analogues, such as meta-iodobenzylguanidine (MIBG), that bind to this transporter would selectively target these neuroblastoma tumor cells [33]. Approximately 90% of children with neuroblastoma have tumors that are detectable by radiolabeled MIBG imaging, and infusion of radiolabeled MIBG, therefore, allows for targeted delivery of radiation therapy directly to sites of active disease. Initial studies of ^131^I-MIBG used therapeutically in children with recurrent neuroblastoma showed overall response rates of 21–47% [34,35,36]. Although useful disease palliation and responses were seen in approximately one-third of patients, clinical benefit was often only temporary [37]. Subsequent studies used MIBG treatment followed by ASCR, as dose escalation was often associated with significant hematopoietic toxicity, particularly in heavily pretreated patients [38]. In a large multi-institutional phase II study, 36% of children with either relapsed or refractory neuroblastoma who were treated with increasing doses of ^131^I-MIBG had either complete or partial responses, while another 34% of patients demonstrated stable disease with a median time to progression of 6 months [39]. A subsequent retrospective analysis of 218 patients treated with ^131^I-MIBG therapy at a single institution demonstrated a 27% overall response rate. However, 24% of relapsed patients had progressive disease compared to only 9% of refractory patients, and 39% of relapsed patients had stable disease compared to 59% of refractory patients [40], suggesting patients with refractory neuroblastoma are most likely to benefit from ^131^I-MIBG therapy. The efficacy of ^131^I-MIBG therapy against neuroblastoma has led to subsequent clinical trials incorporating ^131^I-MIBG therapy as a component of frontline treatment for children with high-risk neuroblastoma, and ongoing clinical trials through the New Approaches to Neuroblastoma Therapy (NANT) consortium are evaluating the efficacy of additional anticancer agents combined with ^131^I-MIBG therapy (NCT02035137) in children with relapsed and refractory neuroblastoma. Despite the established efficacy of ^131^I-MIBG therapy in children with relapsed neuroblastoma, however, only a limited number of institutions have the resources to support the administration of radioactive iodine to pediatric patients, and the limited availability and accompanying need for available stem cells for ASCR remain major challenges to the more widespread use of ^131^I-MIBG therapy.

Prior studies have also shown that 77–89% of neuroblastoma cells express the somatostatin receptor (SSTR) [41,42,43], suggesting that agents targeting the SSTR may also be effective for neuroblastoma therapy. In adults with neuroendocrine tumors (NETs), peptide receptor radionuclide therapy (PRRT) with high-activity ^111^In-, ^177^Lu-, and ^90^Y-labeled somatostatin analogues has been successfully used [44,45]. In a single-institution study of PRRT, four children with relapsed neuroblastoma received 17 cycles of palliative PRRT using either ^111^In-DOTATATE, ^177^Lu-DOTATATE, or ^90^Y-DOTATATE. All four had objective responses, with two long-term survivors [46], supporting ongoing clinical trials.

## 3. Molecularly Targeted Therapy

While regimens incorporating chemotherapy and radiotherapy with ^131^I-MIBG have demonstrated efficacy in the treatment of children with relapsed and refractory neuroblastoma, treatment options for children who suffer from subsequent disease relapses and progression remain limited. However, ongoing investigations exploring the molecular mechanisms underlying the etiology and pathogenesis of neuroblastoma have identified a number of novel therapeutic targets, and several novel targeted agents have been shown to be highly active in preclinical models of neuroblastoma. Many of these agents have been tested or are currently being evaluated in early phase clinical trials for children with relapsed and refractory neuroblastoma (Table 1). Although untreated neuroblastoma tumors have a relative paucity of mutations in therapeutically relevant gene targets [47,48], relapsed neuroblastoma tumors have been found to have a number of actionable mutations [8,9], and initial clinical trials employing therapies selected based on genomic alterations identified in individual patient tumors have demonstrated both feasibility and efficacy, suggesting that molecularly targeted therapy and individualized treatment may lead to increased rates of response and improved outcomes for children with relapsed and refractory neuroblastoma.

### 3.1. Anaplastic Lymphoma Kinase (ALK)

Early efforts to identify gene mutations and other genetic aberrations in neuroblastoma tumors that could serve as therapeutic targets focused on familial neuroblastoma, which accounts for approximately 2% of all patients with neuroblastoma. Initial efforts determined that the majority of these cases are associated with activating germline mutations in the anaplastic lymphoma kinase (*ALK*) gene [49,50,51,52], and subsequent studies identified *ALK* gene mutations or gene amplifications in up to 15% of sporadic high-risk neuroblastoma tumors [49,53]. High-risk neuroblastoma tumors were also found to have increased *ALK* gene expression when compared to low-risk tumors [54], further suggesting a potential role for ALK inhibitors in neuroblastoma therapy. In a subsequent phase I trial, 79 children were enrolled and treated with the ALK inhibitor crizotinib, including 34 with neuroblastoma, 11 of which had known *ALK* mutations [55]. Despite an objective tumor response rate of 67% in children with other tumors with *ALK* mutations, only 1 of 11 children with neuroblastoma with *ALK* mutations (9%) demonstrated an objective response, suggesting that ALK inhibitors will likely need to be combined with other therapies for maximal benefit. Initial studies have identified synergistic combinations of ALK inhibitors with mTOR inhibitors [56] and with CDK4/6 inhibitors [57], and these combinations may serve to overcome some of the limitations of single-agent ALK inhibitor treatment for neuroblastoma. Additionally, novel second-generation ALK inhibitors, such as lorlatinib (PF06463922), ceritinib (LDK378), and ensartinib, that are effective against the crizotinib-resistant ALK^F1174L^ mutant [58,59] are currently being evaluated in clinical trials for children with neuroblastoma (NCT01742286, NCT03107988, NCT03213652), with early results showing responses to ceritinib in six of nine patients with anaplastic large cell lymphoma (ALCL) and myofibroblastic tumors with *ALK* gene aberrations. To date, one patient with relapsed neuroblastoma with an ALK^F1174L^ mutation had shrinkage of a retroperitoneal mass but concurrently experienced central nervous system (CNS) disease progression [60], suggesting that higher doses may be required to achieve adequate levels in neuroblastoma sanctuary sites such as the CNS.

### 3.2. Aurora A Kinase

Additional efforts to identify novel targets in neuroblastoma tumors have identified a critical role for mitotic spindle regulation in neuroblastoma pathogenesis, suggesting that regulators of the mitotic spindle represent potential therapeutic targets. Aurora A kinase represents one such potential target and is essential for appropriate completion of mitosis through regulation of the mitotic checkpoint complex [61]. Aberrant overexpression of aurora A kinase leads to tumor cell resistance to apoptosis and genomic instability [62], and, in neuroblastoma tumors, aurora A kinase expression correlates with high-risk disease and advanced tumor stage [63,64]. Inhibitors of aurora A kinase were shown to block neuroblastoma cell growth and to increase neuroblastoma cell responses to chemotherapy [63], and, in initial phase I trials, children with relapsed neuroblastoma treated with the aurora A kinase inhibitor MLN8237 (alisertib), both alone and in combination with irinotecan and temozolomide, demonstrated clinical responses [65,66]. More recent studies have identified polo-like kinase 4 (PLK4) as a potential target in neuroblastoma tumor cells [67], further implicating the process of mitotic spindle regulation in neuroblastoma pathogenesis and suggesting that children with relapsed neuroblastoma will benefit from the use of inhibitors of aurora A kinase and PLK4 for treatment.

### 3.3. Ornithine Decarboxylase (ODC1)

Ornithine decarboxylase (ODC1), the rate-limiting enzyme in polyamine synthesis, is frequently deregulated in neuroblastoma tumors [68,69] and represents another potential therapeutic target. ODC inhibitors, such as difluoromethylornithine (DFMO), have been shown to be effective in neuroblastoma preclinical models [70,71,72] and, although single-agent DFMO did not demonstrate efficacy in children with relapsed neuroblastoma in a recent phase I clinical trial [73], more recent studies have demonstrated that extended maintenance therapy with DFMO for children with neuroblastoma in second remission results in 2-year overall and event-free survival rates of 54% and 84% [74], respectively, suggesting that ODC1 inhibition is an effective strategy for prolonging survival in these patients. The efficacy of DFMO in combination with other anticancer agents, including cyclophosphamide, topotecan, and celecoxib (NCT02030964) and the proteasome inhibitor bortezomib (NCT02139397), is also currently being evaluated in clinical trials for children with relapsed neuroblastoma, in the hopes of observing synergistic efficacy. 

### 3.4. PI3K/AKT/mTOR

Further studies in neuroblastoma preclinical models have confirmed a role for the PI-3 kinase/AKT/mTOR pathway in neuroblastoma pathogenesis. SF1126 is a pan-PI-3 kinase inhibitor that has been demonstrated to be effective against neuroblastoma in preclinical models [75], suggesting this pathway represents a therapeutic target in neuroblastoma, and clinical trials have been opened to test the safety and tolerability of SF1126 in children with relapsed neuroblastoma (NCT02337309). The AKT inhibitor perifosine has been tested in multiple phase I clinical trials, with 1 complete response and 8 of 27 children with relapsed neuroblastoma demonstrating prolonged stable disease in one phase I study [76], and response rates and disease control rates of 9% and 55% in 11 children with neuroblastoma with measurable disease [77]. Early studies also determined that mTOR inhibitors were effective in neuroblastoma models [78,79], and one of two patients with relapsed neuroblastoma had a CR in initial phase I studies with the mTOR inhibitor temsirolimus [80]. A follow-up phase II study did not meet predetermined efficacy criteria, but 7 of 19 patients with neuroblastoma demonstrated clinical benefit (1 PR, 6 SD) [81], and 2 patients with relapsed neuroblastoma had prolonged stable disease in response to the combination of vinblastine and sirolimus [82], suggesting that a subset of patients may benefit from the inhibition of this pathway. 

### 3.5. Epigenetic Modifications

Epigenetic modifications to DNA involve heritable genomic modifications that are not due to changes in the DNA sequence, including DNA and histone methylation and histone acetylation. These modifications influence chromatin structure and play a key role in regulating gene expression [83]. Epigenetic regulation of gene expression is critical for both normal development and maintenance of tissue-specific gene expression, but aberrant epigenetic mechanisms can contribute to the malignant process, and epigenetic changes contribute to the genomic instability that is a hallmark of cancer [84]. The epigenomic landscape has been shown to contribute to neuroblastoma pathogenesis, leading to early phase clinical trials investigating the safety and efficacy of epigenetic agents [85,86,87], including drugs that target epigenetic writers, such as DNA methyltransferases, as well as epigenetic erasers, such as histone deacetylases [88]. An early phase I study using the demethylating agent decitabine combined with chemotherapy resulted in prolonged stable disease in 5 of 14 patients with relapsed neuroblastoma, although the doses of decitabine were limited due to toxicity [89]. A subsequent study of the combination of the HDAC inhibitor vorinostat combined with bortezomib did not show any responses in the three patients with relapsed neuroblastoma enrolled [90], and further combination studies using vorinostat in children with neuroblastoma are ongoing (NCT02035137, NCT02559778) to identify patient populations that may have improved responses.

Modulation of the epigenetic readers—proteins that recognize and bind to epigenetic chromatin modifications to modulate gene transcription—has recently emerged as a therapeutic strategy for anticancer treatment. Bromodomain and extraterminal domain (BET) proteins are epigenetic readers that regulate gene transcription, and BET inhibitors preferentially bind to super-enhancers—regions of DNA comprising multiple enhancers occupied by transcription factors and regulators of gene transcription that control the expression of genes critical for cell identity, growth, and survival [91]. Further studies have identified critical roles for chromatin structure and super-enhancers in neuroblastoma [92], and BET inhibitors have been shown to be effective against neuroblastoma via targeting of *MYCN* gene expression [93,94]. A number of BET inhibitors are in clinical development and early phase clinical trials, including a novel dual PI3K–BRD4 inhibitor that was effective in neuroblastoma preclinical models [95]. BET inhibitors therefore represent an exciting new class of therapeutic agents for children with relapsed neuroblastoma.

### 3.6. Reactive Oxygen Species (ROS)

Reactive oxygen species (ROS) have been shown to impact a variety of critical intracellular signaling pathways, and prior studies have identified a role for ROS in cancer pathogenesis [96]. However, the role of ROS in neuroblastoma tumors is less clear. Nifurtimox is a nitrofuran derivative that has been used for decades as a primary treatment for Chagas’ disease, a parasitic infection caused by the protozoan *Trypanosoma cruzi* [97,98]. Nifurtimox was shown to inhibit neuroblastoma cell growth both *in vitro* and *in vivo* in preclinical studies, most likely via inhibition of ROS production [99,100], and in early phase clinical trials, patients treated with nifurtimox both as a single agent and in combination with chemotherapy demonstrated responses [101], leading to an ongoing national phase II trial (NCT00601003). A separate study investigating the combination of melphalan with buthionine sulfoximine (BSO) resulted in 6 of 31 patients with relapsed neuroblastoma demonstrating objective responses (5PR, 1MR) [102], and the recent demonstration of synergy between nifurtimox and BSO in other solid tumor models [100] suggests that further testing of the combination of nifurtimox and BSO in neuroblastoma preclinical models is warranted.

### 3.7. Retinoids

Retinoid therapy has been shown to increase EFS rates when used as a component of maintenance therapy for high-risk neuroblastoma [103], and studies have demonstrated potential synergy of retinoids with epigenetic modifying agents [104,105]. An initial phase I study of the combination of the HDAC inhibitor vorinostat with 13-*cis*-retinoic acid had one patient with neuroblastoma who had a CR following treatment [106], and a more recent phase I study of vorinostat in combination with 13-*cis*-retinoic acid for children with relapsed neuroblastoma found no objective responses, but 11 of 29 evaluable patients had SD, with 7 of the 11 receiving at least 11 cycles of therapy [107]. Additionally, the RET tyrosine kinase, which is primarily expressed in cells and tissues derived from the neural crest, has been shown to be required for maturation of the peripheral nervous system, and RET was further shown to be required for neuroblastoma differentiation induced by retinoic acid [108]. RET inhibition has been found to be effective in *in vitro* and *in vivo* preclinical models of neuroblastoma [109,110,111], and a recently opened clinical trial (NCT03611595) will evaluate the efficacy of the combination of RET inhibition and retinoid therapy in children with neuroblastoma and other solid tumors.

### 3.8. Molecularly Guided Therapy

With the rapid advances in our understanding of the biology of cancer, the use of molecular tumor profiling to develop individualized treatment plans is increasingly employed for many adults with a variety of cancers [112]. Although the efficacy of this strategy in children with cancer remains mostly unknown, recently completed clinical trials have evaluated the potential feasibility and efficacy of utilizing molecular and genetic tumor profiling to develop personalized therapy for children with recurrent neuroblastoma. An initial pilot study demonstrated the feasibility of obtaining tumor biopsies from children with relapsed neuroblastoma and then performing DNA sequencing and RNA expression profiling on the relapsed tumor sample, which allowed investigators to generate individualized patient treatment plans in less than 12 days [113]. A follow-up multi-institutional phase I trial employing the same strategy showed that 64% of patients achieved either partial or complete response or disease stabilization for at least one cycle of therapy, with a 7% overall response rate and a progression-free survival time of 59 days [114]. A subsequent single-institution study also demonstrated that the utilization of DNA sequencing data from relapsed tumors to generate prospective treatment plans for children with neuroblastoma was feasible, with nearly half of patients enrolled in the study having potentially actionable genetic findings. Tumor DNA sequencing also led to changes in patient treatment and to genetic counseling for relatives and families of patients found to be at risk of other hereditary disorders [115]. Additional concurrent studies have also demonstrated the feasibility of using genomic data [116] and more comprehensive molecular profiling [117] to identify potential therapeutic targets in children with high-risk, relapsed, or refractory cancers. These results all clearly demonstrate the feasibility of employing molecular profiling to guide individualized treatment strategies for children with neuroblastoma. However, the lack of control groups in these and other studies has limited the ability to assess whether this individualized molecularly targeted treatment resulted in better clinical outcomes for patients when compared with outcomes using standard non-targeted treatment or molecularly targeted agents in an untargeted fashion. Clinical trials to further evaluate the efficacy of molecularly guided, individualized therapy in children with relapsed neuroblastoma and other pediatric solid tumors are currently being developed or are ongoing (NCT02162732, NCT02520713, NCT02638428, NCT03155620, NCT03434262).

## 4. Immunotherapy

Although a number of recent studies have demonstrated the efficacy of various forms of immunotherapy against neuroblastoma and other pediatric solid tumors, the role of immunotherapy in the treatment of patients with relapsed or refractory neuroblastoma is the focus of numerous prior and ongoing studies. Early studies demonstrated promising results for anti-GD2 antibody therapy in children with relapsed neuroblastoma [118,119,120], leading to the evaluation of the efficacy of the chimeric anti-GD2 antibody ch14.18 (dinutuximab) as a component of maintenance therapy for children with high-risk neuroblastoma [121]. Additional studies demonstrated significant efficacy of the mouse anti-GD2 antibody 3F8 in children with relapsed neuroblastoma, with 33% 5-year progression-free survival (PFS) in patients who were treated for relapsed neuroblastoma and achieved either a CR or very good partial response (VGPR) and were then treated with 3F8 plus GM-CSF and 13-*cis*-retinoic acid [10].

### 4.1. Antibody Immunotherapy

Further studies have attempted to expand the use of anti-GD2 antibody therapy in children with relapsed neuroblastoma by employing combinations with other therapies, in addition to the development and evaluation of modified forms of the antibodies themselves. A recent trial combining irinotecan and temozolomide with the anti-GD2 antibody dinutuximab demonstrated promising results, with 9 objective responses (including 5 with CR) among 17 patients receiving the combination [122]. Preliminary data from a follow-up study through the Children’s Oncology Group included data from 53 total eligible patients, with 21 patients (40%) experiencing objective responses, including 11 with CR [123], confirming this combination as a treatment regimen to be considered for children with neuroblastoma at the time of initial relapse. A phase I trial using a humanized version of the ch14.18 antibody with a mutation engineered to reduce side effects (hu14.18K322A) found 6 of 39 enrolled patients had either PR or CR, with 9 additional patients having prolonged SD [124]. A humanized version of the anti-GD2 antibody 3F8 also recently underwent phase I testing, with increased PFS among patients with a higher anti-GD2 antibody titer and reduced overall immunogenicity [125]. In a separate study, 39 patients with recurrent neuroblastoma were treated with the Hu14.18-IL-2 immunocytokine—a fusion protein combining the humanized 14.18 anti-GD2 antibody with IL-2. Of 13 patients with measurable soft tissue neuroblastoma tumors treated with Hu14.18-IL-2, no objective responses were seen, but in those with only MIBG-avid disease or with disease limited to the bone marrow, there were 5 complete responses out of 23 patients [126], suggesting that patients with minimal residual disease are most likely to benefit from this therapy. Further analyses have also shown that mismatches among natural killer (NK) cell KIR/KIR-ligand genotypes and polymorphisms in the Fcγ receptor are also associated with improved responses to anti-GD2 immunotherapy [127,128], suggesting that further strategies for improvement in antibody design and patient selection may result in improved outcomes.

One of the drawbacks to antibody therapy for neuroblastoma is that neuroblastoma tumors use a variety of strategies to evade the host immune response, including downregulation or weak immunogenicity of target antigens and creation of an immune-suppressive tumor environment. With the significant side effects experienced by patients receiving anti-GD2 antibody immunotherapy and with the challenges of antibody administration to patients, a number of alternative immunotherapy strategies are currently under investigation. Immunomodulatory checkpoint inhibitors, such as ipilimumab and nivolumab, have been employed in adult cancers to overcome the immune-suppressive tumor microenvironment. Although ipilimumab was well tolerated in a phase I study for children with relapsed solid tumors, only one patient enrolled in the study had neuroblastoma, and no objective tumor regressions were observed [129]. Ongoing studies evaluating the efficacy of nivolumab with and without added ipilimumab (NCT02304458), with the PD-1 inhibitor pembrolizumab (NCT02332668), and with the anti-PD-L1 antibody atezolizumab (NCT02541604) will hopefully provide more insight to their potential efficacy in children with relapsed neuroblastoma.

Anticancer vaccines have been tested in clinical trials for a number of different tumor types, and an early clinical trial of anti-neuroblastoma vaccine therapy showed the ability to induce an antitumor immune response, although the immune response was insufficient to induce tumor responses or prevent disease progression [130]. A subsequent trial using decitabine combined with a dendritic cell vaccine targeting MAGE-A1, MAGE-A3, and NY-ESO-1 resulted in 1 complete response out of 10 evaluable patients [131], and ongoing cancer vaccine trials include one trial exploring the efficacy of a vaccine using the GD2L and GD3L antigens linked to KLH and administered with the adjuvant OPT-821 and beta-glucan (NCT00911560).

### 4.2. Cell-Based Immunotherapy

CD8+ T lymphocytes play a key role in cell-mediated immunity and, when infused as a form of immunotherapy, offer the advantage of direct tumor targeting that can avoid or overcome the tumor cell strategies to evade the host’s own immune system. Cell therapy with CD8+ T cells has been employed in early phase clinical trials for children with relapsed neuroblastoma, with some notable successes. EBV-specific T lymphocytes engineered to express a chimeric antigen receptor (CAR) directed against GD2 resulted in 3 patients with complete responses out of 11 total treated patients with active disease [132,133]. CAR-T cell persistence was associated with improved responses and longer times to progression in these patients. Next-generation CAR-T cells, using constructs with modified costimulatory domains to regulate T cell activation, are currently being developed [134], and a number of national and international clinical trials are currently ongoing to further explore the efficacy of novel CAR-T cell products targeted against GD2 in children with relapsed neuroblastoma (NCT02239861, NCT02761915, NCT02765243, NCT02919046, NCT03373097). Other trials using CAR-T cells targeted against other cell surface markers, such as CD171, are also currently undergoing testing in clinical trials for children with neuroblastoma (NCT02311621).

Natural killer (NK) cells are another type of cytotoxic lymphocyte that can act in an MHC-unrestricted fashion to target cancer cells. Cell-based immunotherapy using NK cells for children with relapsed neuroblastoma has also been explored, particularly after the report of a child with relapsed neuroblastoma who received haploidentical donor NK cells combined with temozolomide, topotecan, and IL-2 and had a complete response [135]. Ongoing clinical trials are evaluating the efficacy of *ex vivo* expanded haploidentical NK cells infused after haploidentical allogeneic stem cell transplants for children with solid tumors, including neuroblastoma (NCT02100891), and a recently opened clinical trial will explore the efficacy of expanded autologous natural killer T (NKT) cells engineered to express the GD2-specific CAR and IL-15 (NCT03294954). A trial using the hu14.18K322A anti-GD2 antibody combined with chemotherapy, cytokines, and haploidentical NK cells demonstrated a 61% response rate in 13 heavily pretreated patients, with 4 complete responses, 1 very good partial response, and 3 partial responses, in addition to 5 patients with stable disease and 10 of the 13 patients (77%) surviving for at least 1 year [136], demonstrating the efficacy of treatment regimens that combine multiple immunotherapeutic strategies. A number of other trials are investigating the combination of haploidentical NK cells combined with anti-GD2 antibody therapy, including NK cells combined with dinutuxumab and lenalidomide (NCT02573896), humanized 3F8, cyclophosphamide, and IL-2 (NCT02650648), the immunocytokine Hu14.18-IL-2 (NCT03209869); and ch14.18/CHO (NCT03242603). Further studies of this approach are clearly warranted in patients with relapsed neuroblastoma.

## 5. Summary

The treatment of children with relapsed and refractory neuroblastoma remains a challenge, and the outcomes for these children remain poor despite decades of effort by clinicians and scientists. Recent advances in our understanding of the biology of neuroblastoma and of novel strategies to target tumor-specific pathways and antigens have led to a dramatic increase in the number of available treatment options for these patients and give hope that, in the future, novel treatment regimens will increase the responses of tumors to upfront therapy, limit the overall chances of relapse, and, in those hopefully rare cases of relapse, provide safe and effective therapies to eradicate neuroblastoma tumors. Continued development of novel therapies and therapeutic regimens directed against biologically relevant pathways and of novel approaches to harness the therapeutic potential of the innate immune system will provide new treatment strategies to improve the outcomes for these children.

The relative paucity of therapeutically actionable gene mutations has limited the development of individualized treatment regimens for children with neuroblastoma, but as we obtain increased knowledge of other mechanisms of altered gene and protein expression and aberrant function in neuroblastoma tumors, we are likely to identify additional relevant therapeutic targets. Studies to further delineate the critical genetic and proteomic aberrations that either contribute to neuroblastoma pathogenesis or influence neuroblastoma tumor responses to treatment are ongoing, and these aberrations will hopefully lead to the development of individualized patient treatment regimens and also hopefully serve as targets for future drug development. A number of novel therapies directed against recently identified molecular targets are currently being evaluated both in preclinical models and in early phase clinical trials, and established national and international collaborations and cooperative groups will provide opportunities to evaluate these new treatments in carefully controlled clinical trials, leading to more precise and effective therapeutic regimens.

The future holds promise for making considerable advances in our treatment of relapsed neuroblastoma, although continued difficulties in the management of metastatic, widespread relapse and in specific cases of isolated relapses, such as relapse in the CNS, represent ongoing challenges for the future. Although recent results need to be validated in future trials, these results do suggest that we should reconsider our treatment goals for many patients, particularly with the successes of treatments using extended maintenance therapy and immunotherapy for those patients who can achieve disease remission after relapse. Future treatment decisions need to be made based on not only the underlying diagnosis and clinical features of the patient, but also on the molecular features of the tumor itself and the feasibility and availability of tumor-specific treatment. The goals of future clinical and translational research should include identification and validation of the most effective treatment combinations, determination of the most appropriate patients and situations for use of effective therapies, and further delineation of the molecular subgroups of recurrent and refractory neuroblastoma to tailor treatment regimens to the patient populations most likely to benefit. Continued attempts to both develop novel therapeutic agents with efficacy against neuroblastoma tumors and to identify critical intracellular signaling pathways relevant for neuroblastoma pathogenesis and treatment resistance are underway, potentially leading to both individualized and improved treatment and to improved outcomes for children with relapsed and refractory neuroblastoma.

## Figures and Tables

**Table 1:** Current open clinical trials for refractory and relapsed neuroblastoma.

NCT Number	Title	Drug	Molecular Target	Therapeutic Class	Study Enrollment Target	Eligibility Age Range	Primary Site/Group or Sponsor	Status
NCT00089245	Radiolabeled Monoclonal Antibody Therapy in Treating Patients with Refractory, Recurrent, or Advanced CNS or Leptomeningeal Cancer	Intrathecal ^131^I-8H9	B7-H3	Systemic Radiotherapy	120	any	New York, NY (Memorial Sloan Kettering Cancer Center)	Active, recruiting
NCT00107289	Iodine ^131^I Metaiodobenzylguanidine in Treating Patients with Recurrent, Progressive, or Refractory Neuroblastoma or Malignant Pheochromocytoma or Paraganglioma	^131^I-MIBG	NET	Systemic Radiotherapy	80	over 1 year	New York, NY (Memorial Sloan Kettering Cancer Center)	Active, recruiting
NCT00601003	Study of Nifurtimox to Treat Refractory or Relapsed Neuroblastoma or Medulloblastoma	Nifurtimox, cyclophosphamide, topotecan	Reactive Oxygen Species	Systemic Chemotherapy	100	up to 21 years	Beat Childhood Cancer (BCC)	Active, recruiting
NCT00638898	Busulfan, Melphalan, Topotecan Hydrochloride, and a Stem Cell Transplant in Treating Patients with Newly Diagnosed or Relapsed Solid Tumor	Busulfan, melphalan, topotecan	N/A	Systemic Chemotherapy	25	6 months to 40 years	Duarte, CA (City of Hope Cancer Center)	Active, not recruiting
NCT00788125	Dasatinib, Ifosfamide, Carboplatin, and Etoposide in Treating Young Patients with Metastatic or Recurrent Malignant Solid Tumors	Carboplatin, dasatinib, etoposide, ifosfamide	Multiple Kinases (Abl, Src, c-Kit, PDGFRβ)	Molecularly Targeted Therapy	143	1–25 years	Duarte, CA (City of Hope Cancer Center)	Active, not recruiting
NCT00877110	Anti-GD2 3F8 Antibody and Allogeneic Natural Killer Cells for High-Risk Neuroblastoma	Cyclophosphamide, vincristine, topotecan, NK cells, 3F8	GD2	Systemic Chemotherapy plus Immunotherapy	71	any	New York, NY (Memorial Sloan Kettering Cancer Center)	Active, not recruiting
NCT00911560	Bivalent Vaccine with Escalating Doses of the Immunological Adjuvant OPT-821, in Combination with Oral β-glucan for High-Risk Neuroblastoma	Adjuvant OPT-821 in a vaccine containing two antigens (GD2L and GD3L) covalently linked to KLH	GD2L, GD3L	Immunotherapy	215	up to 21 years	New York, NY (Memorial Sloan Kettering Cancer Center)	Active, recruiting
NCT01183884	3F8/GM-CSF Immunotherapy Plus 13-Cis-Retinoic Acid for Consolidation of Second or Greater Remission of High-Risk Neuroblastoma	3F8, GM-CSF, isotretinoin	GD2	Immunotherapy	63	over 18 months	New York, NY (Memorial Sloan Kettering Cancer Center)	Active, not recruiting
NCT01183897	3F8/GM-CSF Immunotherapy Plus 13-Cis-Retinoic Acid for Primary Refractory Neuroblastoma in Bone Marrow	3F8, GM-CSF, isotretinoin	GD2	Immunotherapy	31	over 18 months	New York, NY (Memorial Sloan Kettering Cancer Center)	Active, not recruiting
NCT01331135	Aflac ST0901 CHOANOME—Sirolimus in Solid Tumors (Aflac ST0901)	Sirolimus	mTOR	Molecularly Targeted Therapy	24	up to 30 years	Atlanta, GA (Children’s Healthcare of Atlanta)	Active, not recruiting
NCT01419834	Humanized 3F8 Monoclonal Antibody (Hu3F8) in Patients with High-Risk Neuroblastoma and GD2-Positive Tumors	Hu3F8	GD2	Immunotherapy	74	over 2 years	New York, NY (Memorial Sloan Kettering Cancer Center)	Active, recruiting
NCT01467986	Multimodal Molecular Targeted Therapy to Treat Relapsed or Refractory High-Risk Neuroblastoma	Dasatinib, sirolimus, irinotecan, temozolomide	Multiple Kinases (Abl, Src, c-Kit, PDGFRβ), mTOR	Molecularly Targeted Therapy	114	up to 25 years	Regensburg, Germany (University Hospital Regensburg)	Active, recruiting
NCT01492673	Cyclophosphamide, Topotecan, and Bevacizumab (CTB) in Patients with Relapsed/Refractory Ewing’s Sarcoma and Neuroblastoma	Cyclophosphamide, topotecan, bevacizumab	Angiogenesis	Molecularly Targeted Therapy	9	up to 21 years	New York, NY (Memorial Sloan Kettering Cancer Center)	Active, not recruiting
NCT01576692	Combination Chemotherapy, Monoclonal Antibody, and Natural Killer Cells in Treating Young Patients with Recurrent or Refractory Neuroblastoma	Cyclophosphamide, topotecan, Hu14.18K322A, cisplatin, etoposide, doxorubicin, Vincristine, busulfan, melphalan, natural killer cells, GM-CSF, interleukin-2	GD2	Systemic Chemotherapy plus Immunotherapy	34	up to 21 years	Memphis, TN (St. Jude Children’s Research Hospital)	Active, not recruiting
NCT01601535	Study of MLN8237 in Combination with Irinotecan and Temozolomide	Alisertib (MLN8237), irinotecan, temozolomide	Aurora A Kinase	Molecularly Targeted Therapy	4	1–30 years	New Approaches to Neuroblastoma Therapy (NANT)	Active, not recruiting
NCT01606878	Crizotinib and Combination Chemotherapy in Treating Younger Patients with Relapsed or Refractory Solid Tumors or Anaplastic Large Cell Lymphoma	Crizotinib, cyclophosphamide, topotecan, vincristine, doxorubicin	ALK, c-Met	Systemic Chemotherapy plus Molecularly Guided Therapy	65	1–21 years	Children’s Oncology Group	Active, not recruiting
NCT01625351	A Study of CD45RA+ Depleted Haploidentical Stem Cell Transplantation in Children with Relapsed or Refractory Solid Tumors and Lymphomas	Alemtuzumab, fludarabine, sirolimus, busulfan, melphalan	mTOR	Systemic Chemotherapy plus Immunotherapy	23	2–21 years	Memphis, TN (St. Jude Children’s Research Hospital)	Active, not recruiting
NCT01662804	Humanized 3F8 Monoclonal Antibody (Hu3F8) When Combined with Interleukin-2 in Patients with High-Risk Neuroblastoma and GD2-positive Solid Tumors	Hu3F8, IL-2	GD2	Immunotherapy	14	over 13 months	New York, NY (Memorial Sloan Kettering Cancer Center)	Active, not recruiting
NCT01711554	Lenalidomide and Dinutuximab With or Without Isotretinoin in Treating Younger Patients with Refractory or Recurrent Neuroblastoma	Lenalidomide, dinutuximab, isotretinoin	GD2	Immunotherapy	62	up to 21 years	New Approaches to Neuroblastoma Therapy (NANT)	Active, recruiting
NCT01742286	Phase I Study of LDK378 in Pediatric, Malignancies with a Genetic Alteration in Anaplastic Lymphoma Kinase (ALK)	Ceritinib (LDK378)	ALK	Molecularly Guided Therapy	83	1–17 years	Novartis Pharmaceuticals	Active, not recruiting
NCT01757626	Combination Therapy of Antibody Hu3F8 With Granulocyte- Macrophage Colony Stimulating Factor (GM-CSF) in Patients with Relapsed/Refractory High-Risk Neuroblastoma	Hu3F8 with GM-CSF	GD2	Immunotherapy	224	any	New York, NY (Memorial Sloan Kettering Cancer Center)	Active, recruiting
NCT01804634	A Phase II Trial of Reduced Intensity Conditioning and Haploidentical BMT for High-risk Solid Tumors	Cyclophosphamide, fludarabine, XRT	N/A	Systemic Chemotherapy plus Immunotherapy	20	up to 40 years	Baltimore, MD (Sidney Kimmel Cancer Center at Johns Hopkins)	Active, recruiting
NCT01956669	A Phase II Study of Pazopanib GW786034, NSC# 737754 in Children, Adolescents, and Young Adults with Refractory Solid Tumors	Pazopanib	Multiple Kinases (VEGFR-1, VEGFR-2, VEGFR-3, PDGFRβ, c-kit)	Molecularly Targeted Therapy	154	1–18 years	Children’s Oncology Group	Active, recruiting
NCT02013336	Phase 1 Study of MM-398 Plus Cyclophosphamide in Pediatric Solid Tumors	MM-398 (Irinotecan sucrosofate liposomes), cyclophosphamide	N/A	Systemic chemotherapy	30	1–20 years	South Plains Oncology Consortium	Active, recruiting
NCT02030964	N2012-01: Phase 1 Study of Difluoromethylornithine (DFMO) and Celecoxib with Cyclophosphamide/Topotecan (DFMO)	DFMO, celecoxib, cyclophosphamide, topotecan	ODC	Molecularly Targeted Therapy	30	2–30 years	New Approaches to Neuroblastoma Therapy (NANT)	Active, not recruiting
NCT02034981	Phase 2 Study Assessing Efficacy and Safety of Crizotinib in Patients Harboring an Alteration on ALK, MET or ROS1	Crizotinib	ALK, c-Met	Molecularly Guided Therapy	246	over 1 year	Villejuif, France (Institut Gustave Roussy)	Active, not recruiting
NCT02035137	131I-MIBG Alone VS. 131I-MIBG With Vincristine and Irinotecan VS. 131I-MIBG With Vorinostat (N2011-01)	^131^I-MIBG, vorinostat, vincristine/irinotecan	NET	Systemic radiotherapy plus Chemotherapy	105	2–30 years	New Approaches to Neuroblastoma Therapy (NANT)	Active, recruiting
NCT02076906	MR-guided High Intensity Focused Ultrasound (HIFU) on Pediatric Solid Tumors	MR-HIFU	N/A	N/A	14	up to 30 years	Washington, DC (Children’s National Medical Center)	Active, recruiting
NCT02095132	WEE1 Inhibitor MK-1775 and Irinotecan Hydrochloride in Treating Younger Patients with Relapsed or Refractory Solid Tumors	Irinotecan, AZD1775 (adavosertib)	Wee1	Molecularly Targeted Therapy	154	2–21 years	Children’s Oncology Group	Active, recruiting
NCT02100891	Phase 2 STIR Trial: Haploidentical Transplant and Donor Natural Killer Cells for Solid Tumors (STIR)	NK cells	N/A	Immunotherapy	20	any	Milwaukee, WI (Medical College of Wisconsin)	Active, recruiting
NCT02124772	Study to Investigate Safety, Pharmacokinetic (PK), Pharmacodynamic (PD) and Clinical Activity of Trametinib in Subjects with Cancer or Plexiform Neurofibromas and Trametinib in Combination with Dabrafenib in Subjects with Cancers Harboring V600 Mutations	Trametinib, dabrafenib	MEK	Molecularly Guided Therapy	142	1–17 years	Novartis Pharmaceuticals	Active, recruiting
NCT02139397	Study of DFMO in Combination with Bortezomib for Relapsed or Refractory Neuroblastoma	DFMO, bortezomib	ODC, Proteasome	Molecularly Targeted Therapy	38	up to 21 years	Beat Childhood Cancer (BCC)	Active, recruiting
NCT02162732	Molecular-Guided Therapy for Childhood Cancer		Precision Medicine	Molecularly Guided Therapy	200	13 months to 21 years	Beat Childhood Cancer (BCC)	Active, recruiting
NCT02163356	Fenretinide Lym-X-Sorb + Ketoconazole + Vincristine for Recurrent or Resistant Neuroblastoma (SPOC2013-001)	Fenretinide, ketoconazole, vincristine	N/A	Systemic Chemotherapy	42	up to 30 years	South Plains Oncology Consortium	Active, recruiting
NCT02169609	Safety Study of Dinutuximab Combined with Immunotherapy to Treat Neuroblastoma	Dinutuximab, GM-CSF, isotretinoin, IL-2	GD2	Immunotherapy	25	any	Barcelona, Spain (Hospital Sant Joan de Deu)	Active, not recruiting
NCT02173093	Activated T Cells Armed with GD2 Bispecific Antibody in Children and Young Adults with Neuroblastoma and Osteosarcoma	IL-2, GD2Bi-aATC, GM-CSF	GD2	Immunotherapy	40	13 months to 29 years	Detroit, MI (Children’s Hospital of Michigan)	Active, recruiting
NCT02298348	Sorafenib and Cyclophosphamide/Topotecan in Patients with Relapsed and Refractory Neuroblastoma (N2013-02)	Sorafenib, cyclophosphamide, topotecan	Multiple Kinases (Raf, VEGFR-2, VEGFR-3, c-Kit)	Molecularly Targeted Therapy	18	up to 30 years	New Approaches to Neuroblastoma Therapy (NANT)	Active, not recruiting
NCT02304458	Nivolumab With or Without Ipilimumab in Treating Younger Patients with Recurrent or Refractory Solid Tumors or Sarcomas	Ipilimumab, nivolumab	PD-1, CTLA-4	Immunotherapy	484	1–30 years	Children’s Oncology Group	Active, recruiting
NCT02308527	Activity Study of Bevacizumab with Temozolomide ± Irinotecan for Neuroblastoma in Children	Bevacizumab, temozolomide, irinotecan, topotecan	Angiogenesis	Molecularly Targeted Therapy	160	1–21 years	Cancer Research UK	Active, recruiting
NCT02311621	Engineered Neuroblastoma Cellular Immunotherapy (ENCIT)-01	Anti-CD171 CAR-T cells	CD171	Immunotherapy	40	18 months to 26 years	Seattle, WA (Seattle Children’s Hospital)	Active, recruiting
NCT02332668	A Study of Pembrolizumab (MK-3475) in Pediatric Participants with an Advanced Solid Tumor or Lymphoma (MK-3475-051/KEYNOTE-051)	Pembrolizumab	PD-1	Immunotherapy	310	6 months to 17 years	Merck Sharp & Dohme Corp.	Active, recruiting
NCT02343718	Vinblastine and Temsirolimus in Pediatric Patients with Recurrent or Refractory Lymphoma or Solid Tumours Including CNS Tumours	Temsirolimus, vinblastine	mTOR	Molecularly Targeted Therapy	7	1–18 years	Toronto, ON (Hospital for Sick Children)	Active, not recruiting
NCT02378428	MIBG Therapy for Patients with MIBG Avid Tumors (MIBG)	^131^I-MIBG	NET	Systemic Radiotherapy	65	1–40 years	Columbus, OH (Nationwide Children’s Hospital)	Active, recruiting
NCT02390843	Simvastatin with Topotecan and Cyclophosphamide in Relapsed and/or Refractory Pediatric Solid and CNS Tumors (AflacST1402)	Simvastatin, cyclophosphamide, topotecan	HMG-coA Reductase	Systemic Chemotherapy	36	1–29 years	Atlanta, GA (Children’s Healthcare of Atlanta)	Active, recruiting
NCT02441088	Theranostics: 68GaDOTATOC and 90YDOTATOC (PRRT)	^90^Y-DOTA-tyr3-Octreotide (^90^Y-DOTATOC)	SSTR	Systemic Radiotherapy	25	over 6 months	Iowa City, IA (University of Iowa)	Active, not recruiting
NCT02452554	Lorvotuzumab Mertansine in Treating Younger Patients with Relapsed or Refractory Wilms Tumor, Rhabdomyosarcoma, Neuroblastoma, Pleuropulmonary Blastoma, Malignant Peripheral Nerve Sheath Tumor, or Synovial Sarcoma	Lorvotuzumab mertansine (IMGN-901)	CD56	Immunotherapy	114	1–30 years	Children’s Oncology Group	Active, not recruiting
NCT02508038	TCRαβ+/CD19+ Depleted Haploidentical HSCT + Zoledronate	Zoledronate, TCRαβ+/CD19+ depleted Haploidentical HSCT	N/A	Immunotherapy	21	7 months to 21 years	Madison, WI (University of Wisconsin)	Active, recruiting
NCT02520713	The iCat2, GAIN (Genomic Assessment Informs Novel Therapy) Consortium Study		Precision Medicine	Molecularly Guided Therapy	825	up to 30 years	Boston, MA (Dana-Farber Cancer Institute)	Active, recruiting
NCT02536183	A Phase I Study of Lyso-thermosensitive Liposomal Doxorubicin and MR-HIFU for Pediatric Refractory Solid Tumors	MR-HIFU hyperthermia + lyso-thermosensitive liposomal doxorubicin	N/A	Systemic Chemotherapy	34	up to 30 years	Washington, DC (Children’s National Medical Center)	Active, recruiting
NCT02541604	A Study to Evaluate the Safety, Tolerability, Pharmacokinetics, Immunogenicity, and Preliminary Efficacy of Atezolizumab (Anti-Programmed Death-Ligand 1 [PD-L1] Antibody) in Pediatric and Young Adult Participants with Solid Tumors	Atezolizumab	PD-L1	Immunotherapy	90	up to 30 years	Hoffmann-La Roche	Active, not recruiting
NCT02557854	HIFU Hyperthermia with Liposomal Doxorubicin (DOXIL) for Relapsed or Refractory Pediatric and Young Adult Solid Tumors	Doxil + MR-HIFU Hyperthermia	N/A	Systemic Chemotherapy	14	1–40 years	Dallas, TX (University of Texas Southwestern Medical Center)	Active, recruiting
NCT02573896	Immunotherapy of Relapsed Refractory Neuroblastoma with Expanded NK Cells	NK cells, dinutuximab, lenalidomide	GD2	Immunotherapy	24	1 month to 30 years	New Approaches to Neuroblastoma Therapy (NANT)	Active, not yet recruiting
NCT02574728	Sirolimus in Combination with Metronomic Chemotherapy in Children with Recurrent and/or Refractory Solid and CNS Tumors	Sirolimus, celecoxib, etoposide, cyclophosphamide	mTOR	Molecularly Targeted Therapy	60	1–30 years	Atlanta, GA (Children’s Healthcare of Atlanta)	Active, recruiting
NCT02624388	Study of Genistein in Pediatric Oncology Patients (UVA-Gen001) (UVA-Gen001)	Genistein	N/A	Systemic Chemotherapy	50	1–21 years	Charlottesville, VA (University of Virginia)	Active, recruiting
NCT02630043	Trial of Tolcapone With Oxaliplatin for Neuroblastoma	Tolcapone, oxaliplatin	N/A	Systemic Chemotherapy	21	up to 21 years	Beat Childhood Cancer (BCC)	Active, recruiting
NCT02638428	Genomics-Based Target Therapy for Children with Relapsed or Refractory Malignancy	Ifosfamide, carboplatin, etoposide, axitinib, crizotinib, dasatinib, erlotinib, everolimus, imatinib, pazopanib, ruxolitinib, sorafenib, vandetanib, vemurafenib, trastuzumab, pazopanib, sorafenib	Precision Medicine	Molecularly Guided Therapy	90	up to 18 years	Seoul, Korea (Samsung Medical Center)	Active, recruiting
NCT02639546	Safety and Pharmacokinetics of Cobimetinib in Pediatric and Young Adult Participants with Previously Treated Solid Tumors (iMATRIXcobi)	Cobimetinib	MEK	Molecularly Targeted Therapy	50	6 months to 30 years	Hoffmann-La Roche	Active, recruiting
NCT02641314	Metronomic Treatment in Children and Adolescents with Recurrent or Progressive High Risk Neuroblastoma	Propranolol, celecoxib, cyclophosphamide, vinblastine, etoposide	N/A	Systemic Chemotherapy	26	2–20 years	Cologne, Germany (University of Cologne)	Active, recruiting
NCT02644460	Abemaciclib in Children with DIPG or Recurrent/Refractory Solid Tumors (AflacST1501)	Abemaciclib (LY2835219)	CDK4/6	Molecularly Targeted Therapy	50	2–25 years	Atlanta, GA (Children’s Healthcare of Atlanta)	Active, recruiting
NCT02650401	Study of RXDX-101 in Children with Recurrent or Refractory Solid Tumors and Primary CNS Tumors, With or Without TRK, ROS1, or ALK Fusions	RXDX-101 (Entrectinib)	TRK, ROS1, ALK	Molecularly Guided Therapy	190	2–22 years	Hoffmann-La Roche	Active, recruiting
NCT02650648	Humanized Anti-GD2 Antibody Hu3F8 and Allogeneic Natural Killer Cells for High-Risk Neuroblastoma	Hu3F8, cyclophosphamide, NK cells, IL-2	GD2	Immunotherapy	36	any	New York, NY (Memorial Sloan Kettering Cancer Center)	Active, recruiting
NCT02743429	Phase II Study of Monoclonal Antibody ch14.18/CHO Continuous Infusion in Patients with Primary Refractory or Relapsed Neuroblastoma	ch14.18/CHO	GD2	Immunotherapy	40	1–21 years	Greifswald, Germany (University Medicine Greifswald)	Active, recruiting
NCT02748135	A Two-Part Study of TB-403 in Pediatric Subjects with Relapsed or Refractory Medulloblastoma	TB-403	N/A	Systemic Chemotherapy	36	6 months to 18 years	Beat Childhood Cancer (BCC)	Active, recruiting
NCT02761915	A Cancer Research UK Trial of Anti-GD2 T-cells (1RG-CART)	Anti-GD2 CAR-T cells, cyclophosphamide, fludarabine	GD2	Immunotherapy	27	over 1 year	London, UK (University College London)	Active, recruiting
NCT02765243	Anti-GD2 4th Generation CART Cells Targeting Refractory and/or Recurrent Neuroblastoma	Anti-GD2 CAR-T cells	GD2	Immunotherapy	30	1–14 years	China (Zhujiang Hospital)	Active, recruiting
NCT02780128	Next Generation Personalized Neuroblastoma Therapy (NEPENTHE)	Ceritinib, trametinib, HDM201, ribociclib	ALK, RAS-MAPK, p53	Molecularly Guided Therapy	105	1–21 years	Philadelphia, PA (Children’s Hospital of Philadelphia)	Active, recruiting
NCT02813135	European Proof-of-Concept Therapeutic Stratification Trial of Molecular Anomalies in Relapsed or Refractory Tumors (ESMART)	Ribociclib, topotecan, temozolomide, everolimus, AZD1775, carboplatin, olaparib, irinotecan, AZD2014, nivolumab, Vistusertib, selumetinib, enasidenib, lirilumab	Precision Medicine	Molecularly Guided Therapy	397	up to 18 years	Villejuif, France (Institut Gustave Roussy)	Active, recruiting
NCT02909777	Trial of CUDC-907 in Children and Young Adults with Relapsed or Refractory Solid Tumors, CNS Tumors, or Lymphoma	CUDC-907 (fimepinostat)	PI3K, HDAC	Molecularly Targeted Therapy	44	1–21 years	Boston, MA (Dana-Farber Cancer Institute)	Active, recruiting
NCT02919046	Study Evaluating the Efficacy and Safety With CAR-T for Relapsed or Refractory Neuroblastoma in Children	Anti-GD2 CAR-T cells	GD2	Immunotherapy	22	1–14 years	China (Nanjing Children’s Hospital)	Active, recruiting
NCT02982941	Enoblituzumab (MGA271) in Children with B7-H3-expressing Solid Tumors	Enoblituzumab (MGA271)	B7-H3	Immunotherapy	112	1–35 years	MacroGenics	Active, recruiting
NCT02998983	Racotumomab in Patients with High risk Neuroblastoma	Racotumomab	N-glycolil (NGc) GM3 (NGcGM3)	Immunotherapy	39	1–12 years	Buenos Aires, Argentina (Hospital Universitario Austral)	Active, recruiting
NCT03107988	Study of Lorlatinib (PF-06463922)	Lorlatinib, cyclophosphamide, topotecan	ALK, ROS1	Molecularly Targeted Therapy	40	1–90 years	New Approaches to Neuroblastoma Therapy (NANT)	Active, recruiting
NCT03155620	Targeted Therapy Directed by Genetic Testing in Treating Pediatric Patients with Relapsed or Refractory Advanced Solid Tumors, Non-Hodgkin Lymphomas, or Histiocytic Disorders (Pediatric MATCH)	Palbociclib, selumetinib, ensartinib, vemurafenib, olaparib, larotrectinib, LY3023414, erdafitinib	Precision Medicine	Molecularly Guided Therapy	49	1–21 years	Children’s Oncology Group	Active, recruiting
NCT03189706	Pilot Study of Chemoimmunotherapy for High-Risk Neuroblastoma	Hu3F8, irinotecan/temozolomide, GM-CSF	GD2	Immunotherapy	20	any	New York, NY (Memorial Sloan Kettering Cancer Center)	Active, recruiting
NCT03209869	Treatment of Relapsed or Refractory Neuroblastoma with Expanded Haploidentical NK Cells and Hu14.18-IL2	Hu14.18-IL2, NK cells	GD2	Immunotherapy	6	7 months to 21 years	Madison, WI (University of Wisconsin)	Active, recruiting
NCT03236857	A Study of the Safety and Pharmacokinetics of Venetoclax in Pediatric and Young Adult Patients with Relapsed or Refractory Malignancies	Venetoclax	BCL-2	Molecularly Targeted Therapy	135	up to 25 years	AbbVie	Active, recruiting
NCT03242603	Immunotherapy of Neuroblastoma Patients Using a Combination of Anti-GD2 and NK Cells (NKEXPGD2)	Haploidentical NK cells, ch14.18/CHO	GD2	Immunotherapy	5	6 months to 25 years	Singapore (National University Hospital)	Active, recruiting
NCT03273712	Dosimetry-Guided, Peptide Receptor Radiotherapy (PRRT) With 90Y-DOTA-tyr3-Octreotide (90Y-DOTATOC)	^90^Y-DOTA-tyr3-Octreotide (^90^YDOTATOC)	SSTR	Systemic Radiotherapy	20	6 months to 90 years	Iowa City, IA (University of Iowa)	Active, recruiting
NCT03275402	131I-burtomab Radioimmunotherapy for Neuroblastoma Central Nervous System/Leptomeningeal Metastases	^131^I-burtomab (8H9)	B7-H3	Systemic Radiotherapy	32	up to 18 years	Y-mAbs Therapeutics	Active, not yet recruiting
NCT03294954	GD2 Specific CAR and Interleukin-15 Expressing Autologous NKT Cells to Treat Children with Neuroblastoma (GINAKIT2)	NKT cells, cyclophosphamide, fludarabine	GD2	Immunotherapy	24	1–21 years	Houston, TX (Baylor College of Medicine)	Active, recruiting
NCT03332667	MIBG With Dinutuximab	^131^I-MIBG dinutuximab	NET, GD2	Systemic Radiotherapy plus Immunotherapy	24	1–30 years	New Approaches to Neuroblastoma Therapy (NANT)	Active, not yet recruiting
NCT03373097	Anti-GD2 CAR T Cells in Pediatric Patients Affected by High Risk and/or Relapsed/Refractory Neuroblastoma	Anti-GD2 CAR-T cells	GD2	Immunotherapy	42	1–18 years	Italy (Bambino Gesù Hospital and Research Institute)	Active, recruiting
NCT03434262	SJDAWN: St. Jude Children’s Research Hospital Phase 1 Study Evaluating Molecularly-Driven Doublet Therapies for Children and Young Adults with Recurrent Brain Tumors	Ribociclib, gemcitabine, trametinib, sonidegib	Precision Medicine	Molecularly Guided Therapy	108	1–39 years	Memphis, TN (St. Jude Children’s Research Hospital)	Active, recruiting
NCT03458728	Safety, Tolerability, Efficacy and Pharmacokinetics of Copanlisib in Pediatric Patients	Copanlisib (BAY806946)	PI3K	Molecularly Targeted Therapy	130	6 months to 21 years	Bayer Pharma	Active, recruiting
NCT03478462	Dose Escalation Study of CLR 131 in Children and Adolescents with Relapsed or Refractory Malignant Brain Cancer, Neuroblastoma, Rhabdomyosarcoma, Ewings Sarcoma, and Osteosarcoma	CLR 131	N/A	Systemic Radiotherapy	30	2–21 years	Madison, WI (University of Wisconsin)	Active, not yet recruiting
NCT03507491	Nab-paclitaxel in Combination with Gemcitabine for Pediatric Relapsed and Refractory Solid Tumors	Nab-paclitaxel, gemcitabine	N/A	Systemic Chemotherapy	24	6 months to 30 years	Atlanta, GA (Children’s Healthcare of Atlanta)	Active, not yet recruiting
NCT03561259	A Study of Therapeutic Iobenguane (131I) for High-Risk Neuroblastoma at the Time of First Relapse (OPTIMUM)	^131^I-MIBG	NET	Systemic Radiotherapy	65	over 1 year	Jubilant DraxImage Inc.	Active, recruiting

Molecularly Targeted Therapy—Targeted agent used in non-targeted fashion; Molecularly Guided Therapy—Targeted agent used in targeted patient population based on molecular aberration or phenotype; NET—norepinephrine transporter; SSTR—somatostatin receptor; XRT—radiation therapy; ODC—ornithine decarboxylase, HIFU—high-intensity focused ultrasound, HDAC—histone deacetylase; MIBG—meta-iodobenzylguanidine; CAR-T cells—chimeric antigen receptor T cells; ALK—anaplastic lymphoma kinase; NK cells—natural killer cells.
